# NTH1 Is a New Target for Ubiquitylation-Dependent Regulation by TRIM26 Required for the Cellular Response to Oxidative Stress

**DOI:** 10.1128/MCB.00616-17

**Published:** 2018-05-29

**Authors:** Sarah C. Williams, Jason L. Parsons

**Affiliations:** a Cancer Research Centre, Department of Molecular and Clinical Cancer Medicine, University of Liverpool, Liverpool, United Kingdom

**Keywords:** DNA damage, DNA glycosylase, DNA repair, base excision repair, oxidative stress, ubiquitination

## Abstract

Endonuclease III-like protein 1 (NTH1) is a DNA glycosylase required for the repair of oxidized bases, such as thymine glycol, within the base excision repair pathway. We examined regulation of NTH1 protein by the ubiquitin proteasome pathway and identified the E3 ubiquitin ligase tripartite motif 26 (TRIM26) as the major enzyme targeting NTH1 for polyubiquitylation. We demonstrate that TRIM26 catalyzes ubiquitylation of NTH1 predominantly on lysine 67 present within the N terminus of the protein *in vitro*. In addition, the stability of a ubiquitylation-deficient protein mutant of NTH1 (lysine to arginine) at this specific residue was significantly increased in comparison to the wild-type protein when transiently expressed in cultured cells. We also demonstrate that cellular NTH1 protein is induced in response to oxidative stress following hydrogen peroxide treatment of cells and that accumulation of NTH1 on chromatin is exacerbated in the absence of TRIM26 through small interfering RNA (siRNA) depletion. Stabilization of NTH1 following TRIM26 siRNA also causes significant acceleration in the kinetics of DNA damage repair and cellular resistance to oxidative stress, which can be recapitulated by moderate overexpression of NTH1. This demonstrates the importance of TRIM26 in regulating the cellular levels of NTH1, particularly under conditions of oxidative stress.

## INTRODUCTION

The integrity of genomic DNA is continually compromised by reactive oxygen species (ROS) that are generated endogenously through cellular oxidative metabolism. This results in the generation of DNA base oxidation, base loss (apurinic/apyrimidinic [AP] sites), and DNA strand breaks, and the frequency of DNA base damage events has been estimated to be ∼10,000 per cell per day (1). The consequences of the accumulation of DNA damage are genome instability, initiation of mutagenesis, and the development of human disease, including premature aging, neurodegenerative diseases, and cancer. To maintain genome stability, the base excision repair (BER) pathway plays a vital role in excising damaged DNA bases and coordinating their replacement with the correct undamaged nucleotides (2). The first stage of BER consists of recognition and removal of the damaged base by a damage-specific DNA glycosylase, and 11 of these enzymes are known to exist in human cells (3). The major DNA glycosylases involved in the repair of oxidized DNA bases are 8-oxoguanine DNA glycosylase (OGG1), endonuclease III-like protein 1 (NTH1), and endonuclease VIII-like proteins 1 to 3 (NEIL1 to -3). Following excision of the damaged base, repair generally proceeds by an AP endonuclease 1 (APE1)-dependent mechanism (in the cases of OGG1 and NTH1) or an APE1-independent/polynucleotide kinase phosphatase (PNKP)-dependent mechanism (in the cases of NEIL1 to -3) (4). Following both mechanisms of DNA glycosylase action, DNA polymerase β (Pol β) and the DNA ligase IIIα–X-ray cross-complementing protein 1 (Lig IIIα-XRCC1) complex are required to insert the correct nucleotide and seal the nick in the DNA backbone to restore DNA integrity.

It is recognized that the BER pathway is subject to tight regulation by protein posttranslational modifications, such as acetylation, phosphorylation, and ubiquitylation, which control enzymatic activities, localization, and cellular protein levels (5). Most recently, regulation of BER, and indeed DNA repair pathways in general, through the ubiquitin proteasome pathway (UPP) has been shown to be a vital mechanism for controlling cellular protein levels required for the cellular DNA damage response (6). Ubiquitylation-dependent degradation of proteins is catalyzed by the addition of ubiquitin onto specific lysine residues present within the target protein by E3 ubiquitin ligases (E3s). The addition of polyubiquitin chains generated through internal lysine residues present within the ubiquitin protein usually signals for protein degradation by the 26S proteasome. Currently, more than 600 E3s exist in the human genome, each with its own specific protein substrates. Indeed, specific E3s have been shown to regulate the steady-state and DNA damage-induced levels of BER proteins (4, 5). Most recently, we have demonstrated that two E3s, Mcl-1 ubiquitin ligase E3 (Mule) and tripartite motif 26 (TRIM26), are the major cellular enzymes involved in the ubiquitylation-dependent degradation of NEIL1 and that this mechanism is important for modulating the cellular response to ionizing-radiation-induced DNA damage (7). However, the molecular and cellular mechanisms of regulation of other BER enzymes through the UPP, particularly DNA glycosylases and those responsible for responding to cellular oxidative stress, are unclear.

NTH1 is the major DNA glycosylase that excises oxidized pyrimidines from DNA, including 5-hydroxyuracil, 5-hydroxycytosine, cytosine glycol, and thymine glycol (8). The repair of thymine glycol in DNA is particularly important, as it is a mutagenic lesion that significantly blocks the action of replicative DNA polymerases. While *nth1* knockout mice do not show any overt phenotype, mainly due to redundancy with NEIL1 and NEIL2, the repair of oxidative DNA damage in *nth1* knockout mouse embryonic fibroblasts has been shown to be significantly impaired (9, 10). Furthermore, small interfering RNA (siRNA) depletion of *nth1* in TK6 cells causes sensitivity to hydrogen peroxide treatment, whereas cellular resistance was observed following overexpression of NTH1 (11). These data demonstrate the importance of regulating NTH1 protein levels in the cellular response to oxidative stress. In support of this, reduced expression of NTH1 has been observed in prostate (12) and gastric (13) cancer cells, and it is implicated in the development of liver cancer in a rat model (14, 15). This suggests that the regulation of cellular NTH1 is important in suppressing tumor formation. Interestingly, expression of a natural single nucleotide polymorphism of NTH1 (D239Y), identified in approximately 6% of the population, causes sensitivity to oxidative stress, causes accumulation of DNA double-strand breaks and chromosomal aberrations, and induces cellular transformation (16). Cumulatively, these studies demonstrate the importance of NTH1, particularly the maintenance of the cellular protein levels of NTH1, in conserving genome stability. However, the molecular mechanisms that control NTH1 protein levels are currently unknown. Here, we report the purification of TRIM26 and its identification as the major E3 that regulates ubiquitylation-dependent degradation of NTH1 *in vitro* and *in vivo* and that this mechanism is important in controlling the cellular response to oxidative stress.

## RESULTS

### Identification of TRIM26 as the major E3 ubiquitin ligase targeting NTH1 for ubiquitylation.

**FIG 1:**
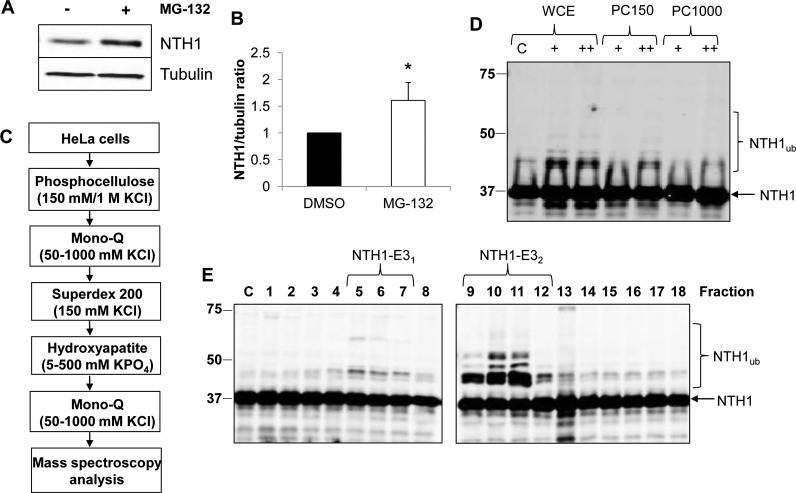
Purification of E3 ubiquitin ligase for NTH1. (A) HCT116 cells grown in 10-cm dishes were treated with the proteasomal inhibitor MG-132 (10 μM) for 8 h. Whole-cell extracts were prepared and analyzed by 10% SDS-PAGE and immunoblotting with the indicated antibodies. (B) NTH1 protein levels in the absence and presence of MG-132 were quantified from the results of at least three independent experiments. Shown is the mean NTH1/tubulin ratio with standard deviation normalized to the DMSO-treated control, which was set to 1.0. *, *P* < 0.05 as analyzed by a two-sample *t* test. (C) Scheme for purification of the E3 ubiquitin ligase for NTH1 from HeLa cell extracts. (D) *In vitro* ubiquitylation of His-tagged NTH1 by HeLa whole-cell extract (WCE) and fractions obtained from phosphocellulose chromatography following low-salt elution (PC150) and high-salt elution (PC1000). + and ++, 2.5-μg and 5-μg fractions, respectively. (E) *In vitro* ubiquitylation of His-tagged NTH1 using fractions from the first ion exchange (Mono Q) chromatography. Ubiquitylation of His-tagged NTH1 (6 pmol) was performed in the presence of E1 activating enzyme (0.7 pmol), ubiquitin (0.6 nmol) (Ub), and all E2 conjugating enzymes (2.5 pmol) and analyzed by 10% SDS-PAGE and immunoblotting using NTH1 antibodies. Lane C, control reactions in the absence of any fraction. Molecular mass markers (in kilodaltons) are indicated on the left, and the positions of unmodified and ubiquitylated NTH1 (NTH1_ub_) are shown.

**FIG 2:**
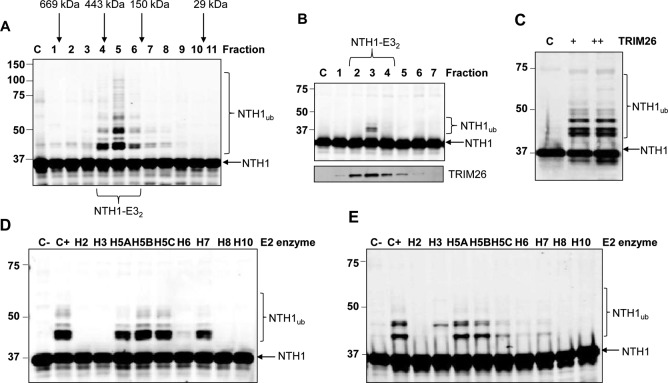
Purification and identification of TRIM26 as the major E3 ubiquitin ligase for NTH1. (A) *In vitro* ubiquitylation of His-tagged NTH1 by fractions obtained from size exclusion (Superdex 200) chromatography. Shown above the blot are the positions of elution of known protein molecular mass standards. (B) *In vitro* ubiquitylation of His-tagged NTH1 by fractions obtained from the final ion exchange (Mono Q) chromatography. Shown below the blot is the alignment of active fractions with TRIM26 protein, as detected by immunoblotting. (C) *In vitro* ubiquitylation of His-tagged NTH1 by His-tagged TRIM26. Lane C, control reaction in the absence of any fraction. + and ++, 19 pmol and 26 pmol TRIM26, respectively. (D and E) Comparison of *in vitro* ubiquitylation of NTH1 by an active fraction purified from HeLa whole-cell extracts (D) or His-tagged TRIM26 (19 pmol) (E) in the presence of individual E2 conjugating enzymes. Control reactions in the absence (C−) or presence (C+) of all E2 enzymes are shown. Unless otherwise indicated, in all the experiments, *in vitro* ubiquitylation of His-tagged NTH1 (6 pmol) was performed in the presence of E1 activating enzyme (0.7 pmol), ubiquitin (0.6 nmol), and E2 conjugating enzymes (2.5 pmol) and analyzed by 10% SDS-PAGE and immunoblotting using NTH1 antibodies. Molecular mass markers (in kilodaltons) are indicated on the left, and the positions of unmodified and ubiquitylated NTH1 (NTH1_ub_) are shown.

**FIG 3:**
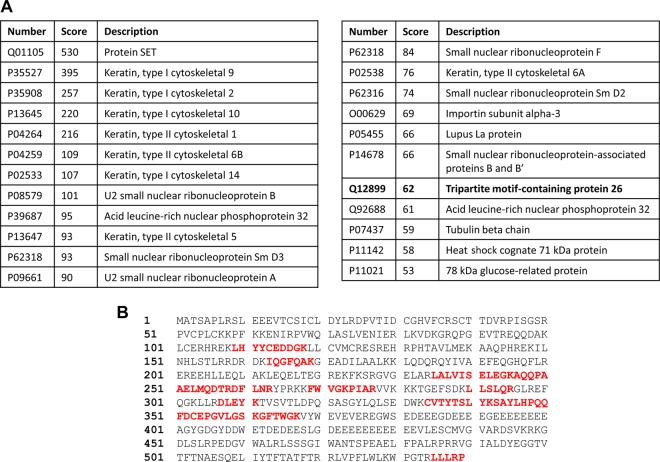
Identification of TRIM26 as the E3 ubiquitin ligase for NTH1 from purified cell extracts. (A) Proteins and mascot scores detected by mass spectrometry derived from an active fraction containing NTH1 ubiquitylation activity from the final 1-ml Mono Q chromatography column purified from HeLa whole-cell extracts. (B) Protein sequence of TRIM26 with the peptide sequences detected by mass spectrometry highlighted in red.

NTH1 is a vital enzyme that repairs oxidized pyrimidines in DNA, particularly the premutagenic lesion thymine glycol, and controls genome stability. Based on this and previous studies documenting ubiquitylation-dependent regulation of BER enzymes, we hypothesized that NTH1 protein is controlled by the UPP, catalyzed by a specific E3(s). Indeed, ubiquitylation of NTH1 has been identified in a proteomic screen (17), although the E3 involved was not identified. In support of our hypothesis, we have shown that the stability of NTH1 in HCT116 cells is statistically significantly increased by ∼1.6-fold in the presence of the proteasomal inhibitor MG-132 (Fig. 1A and B), demonstrating that NTH1 is a target for ubiquitylation-dependent degradation. In order to identify the major E3 involved in this process, we utilized our previously successful and unbiased approach (7, 18–20) employing separation of HeLa cell extracts by column chromatography to generate protein fractions (Fig. 1C). These fractions were then tested for *in vitro* ubiquitylation activity using His-tagged NTH1 as a substrate in the presence of an E1 activating enzyme, 9 different E2 conjugating enzymes, and ubiquitin. Using fractions generated from the first chromatography stage via phosphocellulose separation, we were able to observe weak ubiquitylation activity targeting NTH1. This was revealed by a shift in a protein band of ∼8 kDa (equivalent to the size of ubiquitin) cross-reacting with the NTH1-specific antibodies (ubiquitylated NTH1 [NTH1_ub_]), predominantly in the low-salt elution fraction PC150, as well as in whole-cell extracts (Fig. 1D). The PC150 fraction was subsequently separated by ion exchange (Mono Q) chromatography, which revealed the existence of two ubiquitylation activities for NTH1 (Fig. 1E, fractions 5 to 7 and 9 to 12), one of which (designated NTH1-E3_1_) was extremely weak. We therefore focused on the most significant activity (designated NTH1-E3_2_), which displayed evidence of polyubiquitylation, as revealed by multiple 8-kDa protein shifts, and which was consequently separated by size exclusion (Superdex 200) chromatography. Analysis of protein fractions from the column showed that there was significant polyubiquitylation activity targeting NTH1 that eluted with a molecular mass of 150 to 400 kDa (Fig. 2A, fractions 4 to 6). This activity was further purified by hydroxyapatite chromatography, followed by a final ion exchange (Mono Q) chromatography column, during which time the ubiquitylation activity against NTH1 had been reduced in intensity to a single fraction (Fig. 2B). This highly purified fraction was analyzed by nano-liquid chromatography-tandem mass spectrometry (nano-LC–MS-MS), and the only E3 identified in this fraction was shown to be TRIM26 (Fig. 3A), with sequence coverage of 19% (Fig. 3B). When highly purified protein fractions from the final Mono Q column were analyzed by immunoblotting for the presence of TRIM26, there was a good alignment of the fraction displaying NTH1-E3_2_ activity with that containing the largest amount of TRIM26 protein (Fig. 2B, bottom). This suggests that TRIM26 is the E3 catalyzing ubiquitylation of NTH1 in these highly purified fractions generated from human cell extracts. To confirm this, we used His-tagged TRIM26, which we recently described (7), to show that the purified enzyme can indeed efficiently ubiquitylate NTH1 *in vitro* (Fig. 2C). As mentioned above, throughout the protein purification strategy, *in vitro* ubiquitylation reactions were performed in the presence of 9 different E2 conjugating enzymes. To characterize the activity purified from whole-cell extracts further and to examine the correlation with recombinant TRIM26 activity, separate reactions using reaction mixtures containing each E2 enzyme were performed. This revealed that ubiquitylation of NTH1 by a purified fraction containing NTH1-E3_2_ was dependent on the H5 class of E2 enzymes, as well as H7 (Fig. 2D). In support of our finding that NTH1-E_32_ fractions contain TRIM26, we discovered that His-tagged TRIM26 also ubiquitylates NTH1 using the H5 enzymes and, to a lesser extent, H7 (Fig. 2E). This clearly demonstrates that TRIM26 is a major E3 purified from human cell extracts that is capable of ubiquitylating NTH1 *in vitro*.

### K67 is a major site for ubiquitylation of NTH1 by TRIM26.

**FIG 4:**
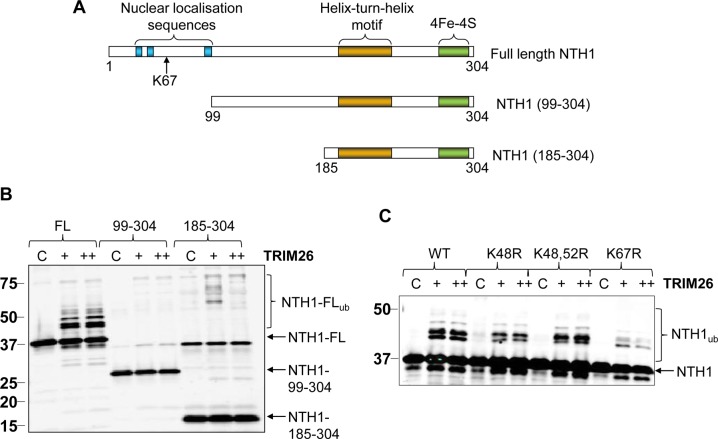
Identification of sites of ubiquitylation within NTH1 by TRIM26. (A) Schematic showing the protein domains within the full-length NTH1 protein and two N-terminal truncations of NTH1 (99 to 304 and 185 to 304). (B) *In vitro* ubiquitylation of His-tagged full-length NTH1 and truncations of NTH1 by His-tagged TRIM26. (C) *In vitro* ubiquitylation of His-tagged NTH1 mutants by His-tagged TRIM26. In all the experiments, *in vitro* ubiquitylation of His-tagged NTH1 (6 pmol) was performed in the presence of E1 activating enzyme (0.7 pmol), H5A E2 conjugating enzyme (2.5 pmol), and ubiquitin (0.6 nmol). + and ++, 19 pmol and 26 pmol TRIM26, respectively. Control reactions in the absence of any His-tagged TRIM26 (lanes C) are shown. All the reactions were analyzed by 10% SDS-PAGE and immunoblotting using NTH1 antibodies. WT, wild type. Molecular mass markers (in kilodaltons) are indicated on the left, and the positions of unmodified and ubiquitylated NTH1 (NTH1_ub_) are shown.

NTH1 is a 304-amino-acid protein containing three putative N-terminal nuclear localization sequences, a helix-2-turn-helix motif, and a C-terminal iron-sulfur cluster (Fig. 4A). The protein sequence also contains 17 lysine residues that are potential targets for ubiquitylation. To identify the sites within NTH1 that are subject to ubiquitylation by TRIM26, we first used a mass spectrometry approach to analyze the products of *in vitro* ubiquitylation reactions. Unfortunately, following digestion with either trypsin or ArgC, we were unable to identify any peptides within NTH1 that were subject to ubiquitylation. Therefore, we created truncation mutants of NTH1 from the N-terminal end of the protein (Fig. 4A) and demonstrated that deletion of the first 98 amino acids of NTH1 was able to significantly inhibit the ubiquitylation of the truncated protein (NTH1-99-304) by TRIM26 *in vitro* in contrast to full-length NTH1 (NTH1-FL), which was efficiently ubiquitylated (Fig. 4B). Additionally, a C-terminally truncated protein containing the last 120 amino acids of NTH1 (NTH1-185-304) was also not ubiquitylated by TRIM26. The N-terminal end of NTH1 contains 5 lysine residues (positions 42, 48, 52, 67, and 75), which are potential targets for ubiquitylation. Therefore, we used site-directed mutagenesis to create site-specific mutants within the full-length NTH1 protein to determine the major ubiquitylation site of TRIM26 *in vitro*. We discovered that mutation of lysine 48, or both lysines 48 and 52, to arginine had only a minor impact on the degree of ubiquitylation of NTH1 by TRIM26 (Fig. 4C). However, mutation of lysine 67 to arginine caused a significant decrease in NTH1 ubiquitylation, suggesting that it is the main residue targeted by TRIM26 *in vitro*.

### TRIM26 regulates newly synthesized and DNA damage-inducible protein levels of NTH1.

**FIG 5:**
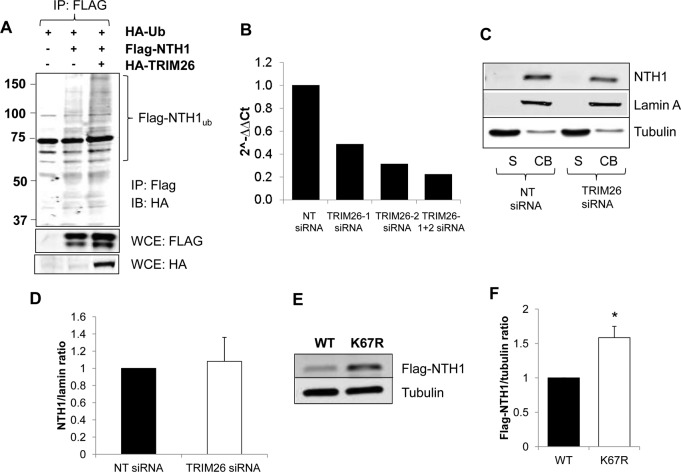
Cellular NTH1 protein levels are regulated by ubiquitylation by TRIM26. (A) HCT116 cells were grown in 10-cm dishes for 24 h to 90% confluence and then treated with Lipofectamine 2000 transfection reagent (10 μl) in the presence of mammalian expression plasmids for HA-tagged ubiquitin (1 μg), Flag-tagged NTH1 (500 ng), and HA-tagged TRIM26 (1 μg) for 24 h. The cells were then treated with MG-132 (10 μM) for 8 h, and whole-cell extracts were prepared and Flag-NTH1 purified, using anti-Flag magnetic beads, from extracts containing equal amounts of total protein. Proteins bound to the beads were analyzed by 10% SDS-PAGE and immunoblotting (IB) with HA antibodies to detect ubiquitylated NTH1. IP, immunoprecipitation. Molecular mass (kilodalton) markers are indicated on the left. (B to D) HCT116 cells were grown in 10-cm dishes for 24 h to 30 to 50% confluence and then treated with Lipofectamine RNAiMax transfection reagent (10 μl) in the presence of 800 pmol NT or TRIM26 siRNA for 72 h. (B) RNA and subsequently cDNA were prepared from cells, and quantitative PCRs using primer pairs for *trim26* and actin were performed. Fold changes in the levels of *trim26* mRNA relative to actin are shown. (C) Proteins were separated by biochemical fractionation, and the soluble (S) and chromatin-bound (CB) fractions were analyzed by 10% SDS-PAGE and immunoblotting with the indicated antibodies. (D) Levels of NTH1 protein relative to lamin A in the chromatin-bound fraction were quantified from the results of at least three independent experiments, and the mean NTH1/lamin A ratio with standard deviation normalized to the NT siRNA-treated control, which was set to 1.0, is shown. (E and F) HCT116 cells were grown in 10-cm dishes for 24 h to ∼90% confluence and then treated with Lipofectamine 2000 transfection reagent (10 μl) in the presence of 250 ng mammalian expression plasmids for Flag-tagged WT or an NTH1 mutant (K67R) for 24 h. (E) Whole-cell extracts were prepared and analyzed by 10% SDS-PAGE and immunoblotting with the indicated antibodies. (F) Levels of Flag-tagged NTH1 proteins relative to tubulin were quantified from the results of at least three independent experiments. Shown is the mean Flag-NTH1/tubulin ratio with standard deviation normalized to that of the WT-NTH1-transfected cells, which was set to 1.0. *, *P* < 0.0005 as analyzed by a one-sample *t* test.

To confirm the role of TRIM26 in the ubiquitylation of NTH1 *in vivo*, we analyzed the effect of combined overexpression of hemagglutinin (HA)-tagged TRIM26 and Flag-tagged NTH1 in the presence of HA-tagged ubiquitin in HCT116 cells. Using a Flag pulldown of NTH1 in the absence of TRIM26, we showed evidence of moderate levels of NTH1 polyubiquitylation, as indicated by a smear of protein cross-reacting with the HA antibodies above 50 kDa (Flag-NTH1_ub_), which is absent in cells expressing HA-tagged ubiquitin only (Fig. 5A, compare lanes 1 and 2). Cellular ubiquitylation of Flag-tagged NTH1 is further enhanced by coexpression with HA-tagged TRIM26 (Fig. 5A, compare lanes 2 and 3), demonstrating that NTH1 is a target for ubiquitylation by TRIM26 in cultured cells. We also examined endogenous NTH1 protein in HCT116 cells following depletion of TRIM26 using siRNA, which we discovered is ∼77% effective, using a combination of two siRNA sequences (Fig. 5B). Cellular fractionation of extracts from nontargeting (NT) control siRNA-treated cells demonstrated that NTH1 protein is almost entirely bound to chromatin and is not in a free soluble form (Fig. 5C, compare lanes 1 and 2). Interestingly, by comparing cells treated with NT control siRNA with cells treated with TRIM26 siRNA, we found that the steady-state levels of NTH1 were not significantly altered in the presence or absence of TRIM26 (Fig. 5C, compare lanes 2 and 4, and D). Given our above-described data demonstrating that transiently expressed NTH1 can be ubiquitylated by TRIM26, this suggests that newly synthesized protein, but not the stable, chromatin-bound form of NTH1, is a target for TRIM26-dependent ubiquitylation. To support this, we analyzed, by plasmid overexpression, the stability of newly synthesized NTH1 in comparison to the K67R mutant of NTH1, which we demonstrated to be deficient in *in vitro* ubiquitylation by TRIM26 (Fig. 4C). We showed that K67R NTH1 protein is statistically significantly more stable (∼1.6-fold) than the wild-type protein (Fig. 5E and F), providing evidence that the stability of newly synthesized NTH1 protein is dependent on lysine 67, the target site for TRIM26 ubiquitylation.

**FIG 6:**
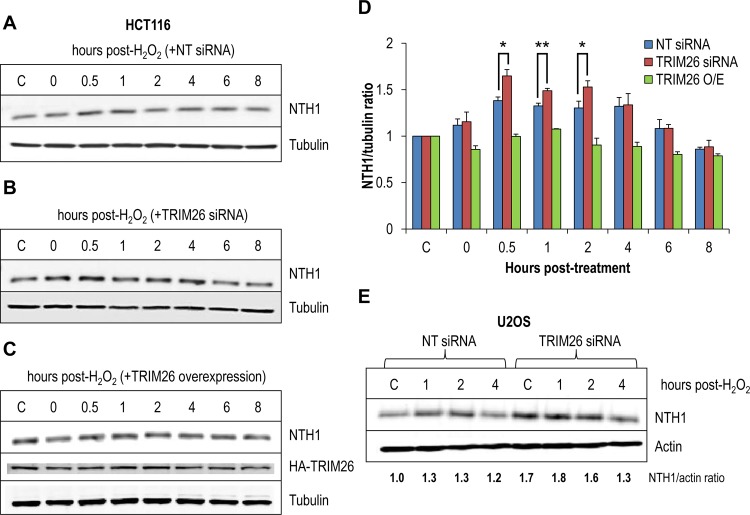
Cellular NTH1 protein levels are induced in response to oxidative stress controlled by TRIM26. (A and B) HCT116 cells were grown in 10-cm dishes for 24 h to 30 to 50% confluence and then treated with Lipofectamine RNAiMax transfection reagent (10 μl) in the presence of 800 pmol NT siRNA (A) or TRIM26 siRNA (Β) for 72 h. (C) HCT116 cells were also grown in 10-cm dishes for 24 h to ∼90% confluence and then treated with Lipofectamine 2000 transfection reagent (10 μl) in the presence of a mammalian expression plasmid for TRIM26 (1 μg) for 24 h. Cells were either left untreated (lane C) or treated with hydrogen peroxide (150 μM for 15 min) and harvested at various time points following incubation. Whole-cell extracts were prepared and analyzed by 10% SDS-PAGE and immunoblotting with the indicated antibodies. (D) Levels of NTH1 protein relative to tubulin were quantified from the results of at least three independent experiments. Shown is the mean NTH1/tubulin ratio with standard error normalized to that of the untreated control, which was set to 1.0. *, *P* < 0.05; **, *P* < 0.02 as analyzed by a one-sample *t* test of ratios at the respective time points comparing NT control siRNA- and TRIM26 siRNA-treated cells. (E) U2OS cells were grown in 10-cm dishes for 24 h to 30 to 50% confluence and then treated with Lipofectamine RNAiMax transfection reagent (10 μl) in the presence of 200 pmol NT siRNA or TRIM26 siRNA for 72 h. Cells were either left untreated (lane C) or treated with hydrogen peroxide (150 μM for 15 min) and harvested at various time points following incubation, and whole-cell extracts were prepared and analyzed by 10% SDS-PAGE and immunoblotting with the indicated antibodies. Levels of NTH1 protein relative to actin were quantified from at least three independent experiments.

We next examined whether TRIM26 plays a role in controlling NTH1 protein levels in the cellular response to DNA damage. HCT116 cells were treated with hydrogen peroxide to induce oxidative stress, and NTH1 protein levels were measured by quantitative immunoblotting at various time points posttreatment. We showed that the levels of NTH1 protein in the presence of an NT control siRNA increased by ∼1.3-fold from 0.5 h posttreatment and remained at this level for up to 4 h before returning to the levels seen in the untreated controls 6 to 8 h post-treatment with hydrogen peroxide (Fig. 6A and D). Following the depletion of TRIM26 using siRNA, there was a modest, but statistically significant, increase in NTH1 protein levels above those seen with the NT siRNA control, particularly at 0.5 to 2 h posttreatment (Fig. 6B and D). It is important to note again that we were able to deplete only ∼75% of TRIM26 protein using siRNA. The residual level of TRIM26 may therefore have suppressed the level of induction of NTH1 observed following treatment with hydrogen peroxide. In support of this, when we overexpressed HA-tagged TRIM26, it suppressed the induction of NTH1 posttreatment, and the levels of NTH1 remained similar to those seen in the untreated controls. We also examined NTH1 protein levels in U2OS cells and observed a similar ∼1.3-fold increase after 1 to 2 h of treatment with hydrogen peroxide in the NT control siRNA cells (Fig. 6E). Interestingly, in those cells, we observed that depletion of TRIM26 by siRNA increased the steady-state protein levels, but also those in response to hydrogen peroxide, 1 to 2 h posttreatment by ∼1.6 to 1.8-fold. Cumulatively, these data suggest that the induction of NTH1 protein, particularly in response to oxidative stress, is closely controlled by TRIM26, which targets NTH1 for ubiquitylation-dependent degradation.

**FIG 7:**
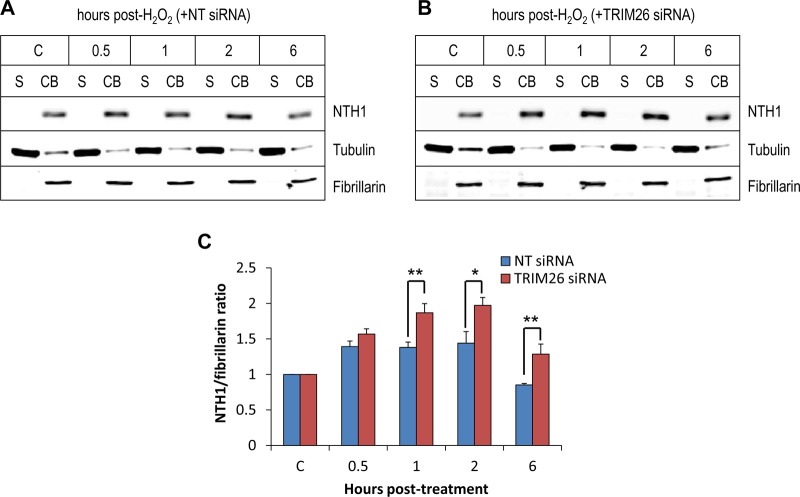
NTH1 protein accumulates on chromatin in response to oxidative stress, which is controlled by TRIM26. (A and B) HCT116 cells were grown in 10-cm dishes for 24 h to 30 to 50% confluence and then treated with Lipofectamine RNAiMax transfection reagent (10 μl) in the presence of 800 pmol NT siRNA (A) or TRIM26 siRNA (Β) for 72 h. Cells were either left untreated (lane C) or treated with hydrogen peroxide (150 μM for 15 min) and harvested at various time points following incubation, and proteins were separated by biochemical fractionation. The soluble (S) and chromatin-bound (CB) fractions were analyzed by 10% SDS-PAGE and immunoblotting with the indicated antibodies. (C) Levels of NTH1 protein relative to fibrillarin in the chromatin-bound fraction were quantified from the results of at least three independent experiments; shown is the mean NTH1/fibrillarin ratio with standard deviation normalized to that of the NT siRNA-treated control, which was set to 1.0. *, *P* < 0.05; **, *P* < 0.02 as analyzed by a one-sample *t* test of ratios at the respective time points comparing NT control siRNA- and TRIM26 siRNA-treated cells.

To investigate further the cellular pool of NTH1 that specifically increases in response to oxidative stress, we fractionated cells into soluble and chromatin-bound fractions at various time points following treatment with hydrogen peroxide. As previously shown, NTH1 protein is almost entirely bound to chromatin and is not in a free soluble form (Fig. 5C). In the presence of an NT control siRNA, NTH1 protein accumulates within the chromatin-bound fraction, particularly between 0.5 and 2 h post-treatment with hydrogen peroxide, and this increase in protein is ∼1.4-fold relative to untreated control cells (Fig. 7A and B). Consistent with our data from whole-cell extracts, depletion of TRIM26 by siRNA causes a significant increase in NTH1 protein levels that are above those observed in NT control siRNA cells. In particular, statistically significantly higher protein levels at 1 to 2 h, but also 6 h, post-treatment with hydrogen peroxide were discovered. This demonstrates that NTH1 protein specifically accumulates on chromatin in the absence of TRIM26.

### The cellular response to oxidative stress is controlled by TRIM26 through the regulation of NTH1.

**FIG 8:**
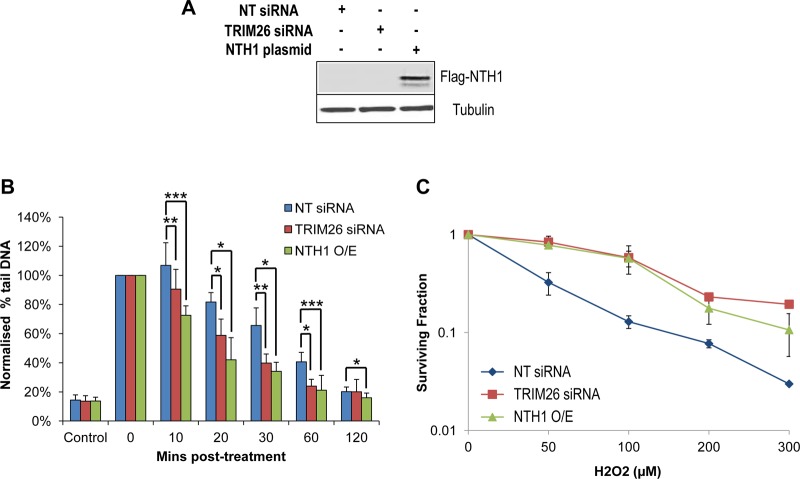
Cellular sensitivity to oxidative stress is controlled by TRIM26 through NTH1 regulation. (A to C) HCT116 cells were grown in 10-cm dishes for 24 h to 30 to 50% confluence and then treated with Lipofectamine RNAiMax transfection reagent (10 μl) in the presence of 800 pmol NT siRNA or TRIM26 siRNA for 72 h. Cells were also treated with Lipofectamine 2000 transfection reagent (10 μl) in the presence of 500 ng mammalian expression plasmid for NTH1 (NTH1 O/E) for 24 h. (A) Whole-cell extracts were prepared and analyzed by 10% SDS-PAGE and immunoblotting with the indicated antibodies. (B) Cells were treated with hydrogen peroxide (12.5 μM), and DNA single-strand breaks and alkali-labile sites were measured at various time points postincubation by the alkaline comet assay. Shown are the percentages of tail DNA with standard deviations from the results of at least three independent experiments. *, *P* < 0.05; **, *P* < 0.02; ***, *P* < 0.01 as analyzed by a one-sample *t* test of percent tail DNA at the respective time points comparing NT control siRNA- and TRIM26 siRNA- or NTH1 O/E-treated cells. (C) Clonogenic survival of HCT116 cells was analyzed following treatment with increasing doses of hydrogen peroxide (0 to 300 μM). Shown are the mean surviving fractions with standard errors from the results of at least three independent experiments. *P* < 2.2 × 10^−16^ (NT siRNA versus TRIM26 siRNA) and *P* < 2.9 × 10^−7^ (NT siRNA versus NTH1 O/E) as analyzed by the CFAssay for R package.

After demonstrating that TRIM26 is required to control the levels of cellular NTH1 on chromatin following hydrogen peroxide treatment, we utilized siRNA depletion of TRIM26 to examine if the mechanism impacts DNA damage repair and overall cell survival. In addition, to mimic the conditions in the absence of TRIM26, where NTH1 protein levels are increased but also to provide evidence that the cellular effects are specifically mediated through NTH1, cells were transfected with a mammalian expression plasmid for NTH1 (Fig. 8A). The level of Flag-tagged NTH1 overexpression was controlled so that it was similar to the level of the endogenous NTH1 protein (see Fig. S1 in the supplemental material). Using an alkaline single cell gel electrophoresis (comet) assay to detect residual DNA single-strand breaks and alkali-labile sites, we showed that DNA damage induced by hydrogen peroxide was repaired steadily over a period of up to 2 h in NT control siRNA-treated cells (Fig. 8B). In contrast, in TRIM26 siRNA-treated cells, DNA damage repair kinetics were accelerated and levels of DNA single-strand breaks and alkali-labile sites were significantly lower than in NT control siRNA-treated cells at 10 to 60 min post-hydrogen peroxide treatment (Fig. 8B, compare blue and red bars). This effect could be mimicked in cells overexpressing NTH1, where the levels of DNA damage were similarly significantly lower than in the NT control siRNA-treated cells at 10 to 120 min post-hydrogen peroxide treatment (Fig. 8B, compare blue and green bars). By analysis of cell survival using clonogenic assays following exposure to increasing amounts of hydrogen peroxide, we further showed that depletion of TRIM26 using siRNA caused a significant increase in resistance of cells to hydrogen peroxide-induced cell killing (Fig. 8C). Importantly, this effect could be phenocopied by overexpression of NTH1. These data suggest that, in the absence of TRIM26, cells treated with oxidative stress have increased DNA damage repair capacity and increased cellular resistance due to an elevation in NTH1 protein levels caused by a lack of ubiquitylation-dependent degradation of the protein.

## DISCUSSION

Our DNA is under constant attack from reactive oxygen species generated through cellular oxidative metabolism, which can cause genome instability. The BER pathway plays a major role in suppressing the accumulation of oxidative DNA damage, and NTH1 is a specific DNA glycosylase that recognizes and excises oxidized pyrimidines, including 5-hydroxyuracil, 5-hydrocytosine, and thymine glycol, from DNA. The importance of NTH1 in the cellular response to oxidative stress is demonstrated by the reduced ability of *nth1* knockout mouse embryonic fibroblasts to repair oxidative DNA damage (9, 10) and by the hypersensitivity of *nth1*-depleted cells to hydrogen peroxide (11). The observation of reduced NTH1 expression in prostate (12) and gastric (13) cancer cells and the importance of the protein in the development of liver cancers (14, 15) also suggest an antitumorigenic role of NTH1. Using an unbiased approach of examining the *in vitro* ubiquitylation activity of protein fractions generated by fractionation of HeLa whole-cell extracts by column chromatography, and utilizing NTH1 as the target protein, we have now isolated and identified TRIM26 as the major E3 in human cell extracts that catalyzes polyubiquitylation of NTH1 *in vitro*. This posttranslational modification occurs predominantly on lysine 67 of NTH1, and when a lysine-to-arginine mutant protein at this specific position is transiently expressed in cells, the mutant protein is significantly more stable than the wild-type protein. We also demonstrated that TRIM26 directly polyubiquitylates NTH1 in cells and that TRIM26 targets newly synthesized NTH1 protein for ubiquitylation-dependent degradation. Furthermore, the absence of TRIM26 through siRNA depletion leads to the accumulation of NTH1 on chromatin in response to oxidative stress, increased oxidative DNA damage repair capacity, and resistance to hydrogen peroxide-induced cell killing.

TRIM proteins are thought to play key roles in innate antiviral immunity, and the proteins also contain a RING finger motif, indicative of E3 activity (21). TRIM26 in particular has been shown to promote ubiquitylation-dependent degradation of the transcription factor IRF3, which reduces interferon beta production and regulates antiviral responses (22). However, we recently demonstrated that NEIL1, a DNA glycosylase particularly associated with excising oxidative DNA damage from single-stranded DNA generated through transcription or replication, is a target for ubiquitylation-dependent degradation by TRIM26, in addition to another E3, Mule (7). This was discovered by using an unbiased approach similar to the one utilized in this study, incorporating fractionated cell extracts with an *in vitro* ubiquitylation assay containing the target protein. Interestingly, the steady-state levels of NEIL1 protein were found to be regulated by both TRIM26 and Mule, although the induction of NEIL1 in response to ionizing radiation was Mule dependent but not TRIM26 dependent. This is in contrast to our current data on the regulation of NTH1 by TRIM26 in HCT116 cells, where we show that depletion of cellular TRIM26 (albeit ∼77% efficient) had no dramatic impact on the steady-state levels of NTH1 but that NTH1 protein significantly accumulated in response to oxidative stress. However, increased steady-state levels of NTH1 were observed in U2OS cells following depletion of TRIM26. The accumulation of NTH1 protein following hydrogen peroxide treatment in NT control siRNA-treated HCT116 and U2OS cells is modest (∼1.3-fold above untreated-control levels), although this is consistent with our previous data examining the regulation of other BER protein levels, including Pol β, PNKP, and NEIL1, in response to oxidative stress (7, 18, 23). This suggests that BER has a limited capacity designed to respond to minor fluctuations in endogenous DNA damage. Therefore, with the continuous oxidative DNA damage that mammalian cells encounter as a consequence of cellular oxidative metabolism or via exogenous sources, including ionizing radiation, BER has evolved as a DNA repair mechanism that is not significantly inducible. It is expected that if cells do receive a significant amount of oxidative DNA damage that is beyond the capacity of BER to repair, then the cells undergo apoptosis. Nevertheless, the further increase in NTH1 protein levels caused by depletion of TRIM26, and particularly our observation that NTH1 accumulates on chromatin, leads to a significantly increased ability of cells to repair oxidative DNA damage and reduces cellular sensitivity to oxidative stress through the modulation of NTH1. This effect can be recapitulated by moderate overexpression of NTH1 alone, suggesting that NTH1 is responsible for these cellular effects. Our data are supported by evidence that overexpression of NTH1 in TK6 cells causes resistance to hydrogen peroxide-induced cell killing, whereas depletion of *nth1* enhanced sensitivity (11). Nevertheless, our new data further support our previous findings of the major impact and importance of TRIM26 in controlling and coordinating the cellular response to DNA damage, but we now provide evidence that this is achieved through the regulation of two DNA glycosylases, NEIL1 and NTH1. Interestingly, there is evidence that TRIM26 may act as a tumor suppressor in hepatocellular carcinoma (24), although whether this is directly related to its role in regulation of NEIL1 and NTH1 is unclear. This may also be associated with the function of TRIM26 in regulation of transcription through IRF3 (22), combined with recent evidence demonstrating that TRIM26 catalyzes ubiquitylation-dependent degradation of the transcription factor IID subunit TAF7 in response to transforming growth factor β (TGF-β) stimulation (25). However, a potential tumor suppressor role for TRIM26 and the mechanism involved require further investigation.

An unresolved question is the precise cellular mechanism that governs the identification of NTH1 and NEIL1 for ubiquitylation-dependent degradation by TRIM26, particularly as NTH1 regulation following oxidative DNA damage occurs largely in a TRIM26-dependent manner. This is in contrast to NEIL1, where we previously observed that depletion of TRIM26 does not alter the induction of NEIL1 protein levels in response to ionizing radiation but does control the steady-state levels of the protein (7). The explanation, in part, may be that NTH1 and NEIL1 appear to have separate cellular roles, where NTH1 is involved in the general repair of oxidized pyrimidines within DNA, which is in contrast to NEIL1, which is mainly associated with repair of single-stranded DNA generated during replication and transcription. Nevertheless, given these two different roles for TRIM26 in the regulation of the two DNA glycosylases, it is entirely feasible that NTH1 and NEIL1 themselves are subject to further regulation by posttranslational modifications that either promote or inhibit ubiquitylation by TRIM26. However, neither NTH1 nor NEIL1 has been previously reported to be modified by acetylation, methylation, phosphorylation, or SUMOylation (summarized in reference 5). Therefore, further investigation is required to reveal any potential cross talk between these modifications and TRIM26-dependent ubiquitylation in the controlled degradation of the proteins. Once this has been established, how these mechanisms and the enzymes controlling them respond to different cellular stresses (e.g., ionizing radiation and oxidative stress) will also require further examination. Second, it is notable that the increased cellular levels of NTH1 protein following oxidative stress in the absence of TRIM26 return to those seen in the untreated controls several hours posttreatment. It is possible that either the residual levels of TRIM26 following siRNA (∼23%) treatment in HCT116 cells are able to eventually degrade NTH1, albeit at a lower rate, or, alternatively, that a second E3 exists that may be able to compensate for the lack of TRIM26. In support of the latter, our initial purification data demonstrated the existence of a second E3 for NTH1 (designated NTH1-E3_1_) that contained significantly reduced *in vitro* ubiquitylation activity for NTH1 versus TRIM26 but that may have a more prominent role in cells. Our initial attempts to purify this E3 have proved unsuccessful, as the protein and/or activity has proved very unstable following further protein fractionation. Nevertheless, to address the two points, it will be necessary to generate multiple knockout cell lines of TRIM26 (e.g., by CRISPR-Cas9 gene editing) and to reexamine the stability of NTH1 and NEIL1 in both the absence and presence of oxidative stress but also to discover alternate strategies for the purification of the additional E3 for NTH1. This will be the focus of our future investigations.

In summary, we have demonstrated that TRIM26 plays a vital role in regulating the cellular protein levels of NTH1 through ubiquitylation-dependent degradation. This, in combination with its function in modulating NEIL1, highlights an important role for TRIM26 in controlling the cellular response to DNA damage.

## MATERIALS AND METHODS

### Materials.

NTH1 (ab70726), TRIM26 (ab89290), and HA (ab9110) antibodies were from Abcam (Cambridge, United Kingdom). Tubulin and Flag antibodies were from Sigma-Aldrich (Gillingham, United Kingdom), and lamin antibodies were from Santa Cruz Biotechnology (Heidelberg, Germany). HeLa cell pellets for protein fractionation by column chromatography were from Cilbiotech (Mons, Belgium). Ubiquitin was purchased from Boston Biochemicals (Cambridge, MA). Bacterial expression plasmids for E1 (UBE1) and 9× E2 (UBCH2, UBCH3, UBCH5A, UBCH5B, UBCH5C, UBCH6, UBCH7, UBCH8, and UBCH10) enzymes were acquired from Addgene (Cambridge, MA). The mammalian expression plasmid for HA-tagged TRIM26 was kindly provided by A. Garcia-Sastre, and a bacterial expression plasmid for His-tagged TRIM26 was generated as we recently described (7). The mammalian expression plasmid for HA-tagged ubiquitin was kindly provided by D. Bohmann. Full-length *nth1* cDNA was recloned by ligation-independent cloning (26) from a bacterial expression plasmid (pET28a) for NTH1 into the pCMV-Tag3a vector for mammalian expression. Site-directed PCR mutagenesis was used to generate site-specific mutants within NTH1. His-tagged TRIM26, NTH1, E1, and E2 enzymes were overexpressed in Rosetta2(DE3)pLysS bacterial cells (Merck-Millipore, Watford, United Kingdom) and purified using HisTrap column chromatography (GE Healthcare, Little Chalfont, United Kingdom). TRIM26 was additionally purified using ion exchange (Mono Q 5/5 GL) chromatography (GE Healthcare, Little Chalfont, United Kingdom).

### Cell culture and RNA interference.

HCT116^p53+/+^ cells were cultured in Dulbecco's modified Eagle medium (DMEM) supplemented with 10% fetal bovine serum, 2 mM l-glutamine, 1× penicillin-streptomycin, and 1× nonessential amino acids at 37°C in 5% CO_2_. For siRNA knockdowns, cells were grown in 10-cm dishes for 24 h to 30 to 50% confluence and treated with 10 μl Lipofectamine RNAiMax transfection reagent (Life Technologies, Paisley, United Kingdom) in the presence of 800 pmol Qiagen AllStars negative-control siRNA (Qiagen, Southampton, United Kingdom), TRIM26 siRNA-1 (5′-CCGGAGAAUUCUCAGAUAA-3′), or TRIM26 siRNA-2 (5′-GAGUCACAGGAACUCAUCU-3′), for a further 72 h. For hydrogen peroxide studies, cells were treated with the stated concentrations for 15 min prior to processing for various assays.

### Quantitative PCR.

RNA was prepared from HCT116^p53+/+^ cells treated with NT control siRNA or TRIM26 siRNA using an RNeasy kit (Qiagen, Crawley, United Kingdom), and cDNA was generated using a GoScript reverse transcription kit (Promega, Southampton, United Kingdom). Quantitative-PCR mixtures containing SYBR Select master mix (Life Technologies, Paisley, United Kingdom) and primer pairs for *trim26* (5′-CCATGGATCTATAGGAGAGCAAG-3′; 5′-CAGCTCCAGCACTCAGTCAA-3′) and actin (5′-AGGCACCAGGGCGTGAT-3′; 5′-CGCCCACATAGGAATCCTTCT-3′) were prepared. Reactions were analyzed using an Applied Biosystems 7500 real-time PCR system (Life Technologies, Paisley, United Kingdom). Δ*C_T_* values were calculated by subtracting threshold cycle (*C_T_*) values for *trim26* from *C_T_* values for actin. ΔΔ*C_T_* values were generated by subtracting Δ*C_T_* values for the NT control siRNA from those for TRIM26 siRNA, and fold changes (2^−ΔΔ*CT*^) were calculated.

### Whole-cell extract preparation and cell fractionation.

Whole-cell extracts were prepared from harvested cell pellets as previously described (7, 27). Briefly, cell pellets were resuspended in one packed cell volume (PCV) of buffer containing 10 mM Tris-HCl (pH 7.8), 200 mM KCl, 1 μg/ml of each protease inhibitor (pepstatin, aprotinin, chymostatin, and leupeptin), 1 mM phenylmethylsulfonyl fluoride (PMSF), and 1 mM *N*-ethylmaleimide (NEM). A further two PCVs of buffer containing 10 mM Tris-HCl (pH 7.8), 600 mM KCl, 40% glycerol, 2 mM EDTA, 0.2% IGEPAL CA-630, 1 μg/ml of each protease inhibitor, 1 mM PMSF, and 1 mM NEM was added, and the extract was mixed thoroughly. The cell suspension was mixed by rotation for 30 min at 4°C and centrifuged at 40,000 rpm at 4°C for 20 min, and the supernatant was collected and stored at −80°C. Typically, 40 μg protein was used for immunoblotting analysis. Cell fractionation generating soluble and chromatin-bound protein fractions was also performed as previously described (7).

### Purification of the E3 ubiquitin ligase from HeLa whole-cell extracts.

HeLa whole-cell extracts were prepared from 20 g HeLa cell pellets and dialyzed against buffer A (50 mM Tris-HCl [pH 8.0], 1 mM EDTA, 5% glycerol, 1 mM dithiothreitol [DTT], and 100 μM phenylmethylsulfonyl fluoride [PMSF]) containing 150 mM KCl. The cell extract was clarified by centrifugation (25,000 rpm for 20 min), filtered through 0.45-μm syringe filters, and added to a 250-ml P-11 phosphocellulose column, and the flowthrough (designated PC-150) was collected. The PC-150 fraction was diluted 2-fold with buffer A to achieve a final concentration of 75 mM KCl and then added to a 20-ml HiLoad Mono Q Sepharose column (GE Healthcare, Little Chalfont, United Kingdom). The column was washed with buffer A containing 50 mM KCl, proteins were eluted into 4-ml fractions using a 400-ml linear gradient from 50 to 1,000 mM KCl, and active fractions were then pooled and concentrated using Amicon Ultra-15 centrifugal-filter units (Millipore, Watford, United Kingdom). The proteins were loaded onto a Superdex 200 HR 10/30 column (GE Healthcare, Little Chalfont, United Kingdom) in buffer A containing 150 mM KCl, and 0.5-ml fractions were collected. Active fractions were pooled and concentrated, and buffer was changed using Amicon Ultra-15 centrifugal-filter units to buffer B (5 mM KPO_4_ [pH 7.0], 5% glycerol, 1 mM DTT, and 100 μM PMSF). The proteins were applied to a 1-ml CHT ceramic hydroxyapatite column (Bio-Rad, Hemel Hempstead, United Kingdom) in buffer B and eluted into 0.5-ml fractions using a linear gradient of 5 to 500 mM KPO_4_. Active fractions were pooled, diluted 10-fold in buffer A, and then loaded onto a Mono Q 5/50 GL column (GE Healthcare, Little Chalfont, United Kingdom) in buffer A containing 50 mM KCl, and the proteins were eluted into 0.5-ml fractions using a linear gradient of 50 to 1,000 mM KCl. After each chromatography stage, the protein fractions were examined for *in vitro* NTH1 ubiquitylation activity, and those displaying significant activity were pooled for the next chromatography step. Proteins present in active fractions from the final Mono Q chromatography were identified by tandem mass spectrometry using a Q Exactive instrument operated in data-dependent positive (electrospray ionization^+^ [ESI^+^]) mode, as recently described (7).

### *In vitro* ubiquitylation assay.

Ubiquitylation reaction mixtures containing 6 pmol His-NTH1, 0.7 pmol glutathione *S*-transferase (GST)–E1 activating enzyme, 2.5 pmol E2 conjugating enzyme (a combination of 9 different E2s, unless otherwise stated), and 0.6 nmol ubiquitin in buffer containing 25 mM Tris-HCl (pH 8.0), 4 mM ATP, 5 mM MgCl_2_, 200 μM CaCl_2_ and 1 mM DTT were incubated in LoBind protein tubes (Eppendorf, Stevenage, United Kingdom) for 1 h at 30°C with agitation. The reactions were halted by the addition of SDS-PAGE sample buffer (25 mM Tris-HCl [pH 6.8], 2.5% β-mercaptoethanol, 1% SDS, 10% glycerol, 1 mM EDTA, 0.05 mg/ml bromophenol blue) and heated for 5 min at 95°C prior to SDS-PAGE and immunoblotting.

### Cellular ubiquitylation assay.

HCT116^p53+/+^ cells were grown in 10-cm dishes for 24 h to ∼90% confluence and then treated with 10 μl Lipofectamine 2000 (Life Technologies, Paisley, United Kingdom) in the presence of mammalian expression plasmids for HA-tagged ubiquitin (1 μg), Flag-tagged NTH1 (500 ng), or HA-tagged ubiquitin (1 μg) for 24 h. The cells were then incubated with the proteasomal inhibitor MG-132 (10 μM) for 8 h and pelleted by centrifugation, and whole-cell extracts were prepared. Equal amounts of protein in the extracts were incubated with 10 μl anti-Flag M2 magnetic beads (Sigma-Aldrich, Gillingham, United Kingdom) for 2 h at 4°C with rotation, and the beads were separated using a magnetic separation rack and washed three times with 500 μl buffer A containing 150 mM KCl. SDS-PAGE sample buffer was added to the beads and heated for 5 min at 95°C prior to SDS-PAGE and immunoblotting using HA antibodies to examine the degree of NTH1 ubiquitylation.

### Immunoblotting.

Protein extracts (typically 40 μg) or *in vitro* ubiquitylation reaction mixtures in SDS-PAGE sample buffer were heated for 5 min at 95°C and separated by 10% Tris-glycine SDS-PAGE. The proteins were transferred onto an Immobilon FL polyvinylidene difluoride (PVDF) membrane (Millipore, Watford, United Kingdom), blocked using Odyssey blocking buffer (Li-Cor Biosciences, Cambridge, United Kingdom), and incubated with the primary antibody diluted in Odyssey blocking buffer with 0.1% Tween 20 overnight at 4°C. The membranes were washed three times with phosphate-buffered saline (PBS) containing 0.1% Tween 20 (5-min washes), incubated with either Alexa Fluor 680- or IR Dye 800-conjugated secondary antibodies for 1 h at room temperature, and again washed three times with PBS containing 0.1% Tween 20. After a final wash with PBS, the proteins were visualized and quantified using the Odyssey image analysis system (Li-Cor Biosciences, Cambridge, United Kingdom).

### Clonogenic assays.

HCT116^p53+/+^ cells grown in 35-mm dishes were treated in the absence and presence of TRIM26 siRNA (800 pmol) using Lipofectamine RNAiMax (Life Technologies, Paisley, United Kingdom) for 48 h. For NTH1 overexpression, cells were treated with 50 ng pCMV-Tag3a-NTH mammalian expression plasmid using Lipofectamine 2000 (Life Technologies, Paisley, United Kingdom) for 24 h. The cells were treated with hydrogen peroxide (0 to 300 μM) for 15 min, trypsinized, and counted, and a defined number were seeded in triplicate into 6-well plates and incubated at 37°C in 5% CO_2_. Note that increasing cell numbers were used for increasing doses of hydrogen peroxide and also that double the numbers of cells were plated for TRIM siRNA to account for cellular plating efficiencies. Colonies were allowed to grow for 7 to 10 days prior to fixing and staining with 6% glutaraldehyde, 0.5% crystal violet for 30 min. The plates were washed and left to air dry overnight, and the colonies were counted using a GelCount colony analyzer (Oxford Optronics, Oxford, United Kingdom). Relative colony formation (surviving fraction) was expressed as colonies per treatment level versus colonies that appeared in the untreated control. Statistical analysis was performed using the CFAssay for R package (28).

### Alkaline comet assay.

The alkaline comet assay, examining DNA repair activities in gel, was performed as described previously (29). Cells were trypsinized and diluted to ∼1 × 10^5^ cells/ml, and 250-μl aliquots of the cell suspension were placed into the wells of a 24-well plate on ice. The cells were treated with hydrogen peroxide (12.5 μM) in suspension for 5 min and then embedded in 1% low-melting-point agarose (Bio-Rad, Hemel Hempstead, United Kingdom) on a microscope slide precoated with 1% normal-melting-point agarose and allowed to set on ice. The slides were placed in a humidified chamber to allow the cells to undergo DNA repair in gel for up to 2 h at 37°C prior to being placed in freshly prepared cell lysis buffer containing 2.5 M NaCl, 100 mM EDTA, 10 mM Tris-HCl, pH 10.5, 1% (vol/vol) dimethyl sulfoxide (DMSO), and 1% (vol/vol) Triton X-100 for at least 1 h at 4°C. The slides were transferred to an electrophoresis tank and incubated in the dark for 30 min in fresh cold electrophoresis buffer (300 mM NaOH, 1 mM EDTA, 1% [vol/vol] DMSO, pH 13) to allow the DNA to unwind. Electrophoresis was performed at 25 V and 300 mA for 25 min, and the slides were neutralized with three 5-min washes with 0.5 M Tris-HCl (pH 8.0) and allowed to air dry overnight. The slides were rehydrated for 30 min in water (pH 8.0), stained for 30 min with SYBR Gold (Life Technologies, Paisley, United Kingdom) diluted 1:20,000 in water (pH 8.0), and allowed to air dry prior to imaging. The cells (50 per slide; 2 slides per time point) were analyzed using Komet 6.0 image analysis software (Andor Technology, Belfast, Northern Ireland). Percent tail DNA values were averaged from the results of at least three independent experiments.

## Supplementary Material

Supplemental material
