# Supplementary material for: NTH1 Is a New Target for Ubiquitylation-Dependent Regulation by TRIM26 Required for the Cellular Response to Oxidative Stress
Source: Mol Cell Biol. 2018 May 29;38(12):e00616-17. doi: 10.1128/MCB.00616-17 (PMC5974432; doi:10.1128/MCB.00616-17)
Supplement: Supplemental material [file supp_38_12_e00616-17__index.html]

Supplemental material 

# NTH1 Is a New Target for Ubiquitylation-Dependent Regulation by TRIM26 Required for the Cellular Response to Oxidative Stress

## Supplemental material

- Supplemental file 1 -

  Fig. S1 (Overexpression of NTH1 in HCT116 cells)

  PDF, 63K
