# Supplementary material for: NTH1 Is a New Target for Ubiquitylation-Dependent Regulation by TRIM26 Required for the Cellular Response to Oxidative Stress
Source: Mol Cell Biol. 2018 May 29;38(12):e00616-17. doi: 10.1128/MCB.00616-17 (PMC5974432; doi:10.1128/MCB.00616-17)
Supplement: Supplemental material [file MCB.00616-17_zmb999101776s1.pdf]

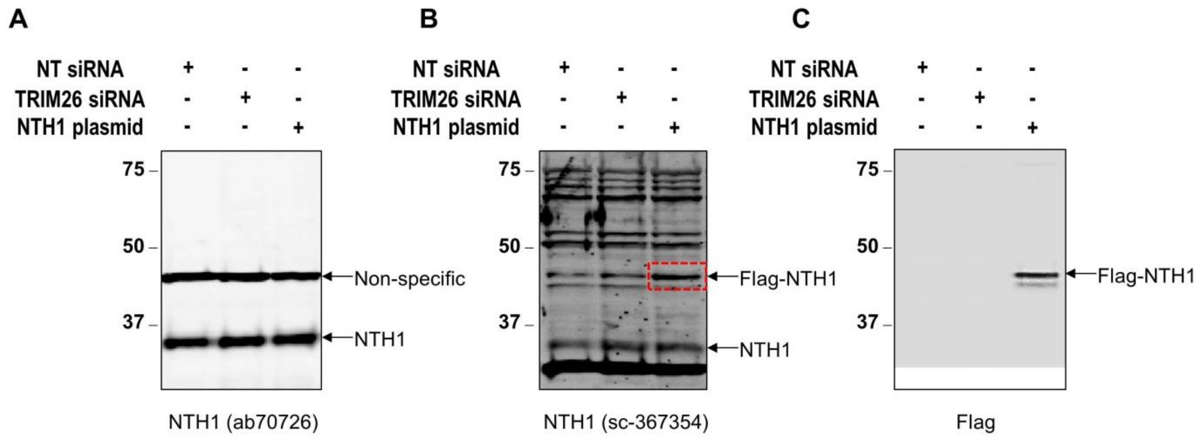

Figure S1. Determination of the level of overexpression of NTH1 in HCT116 cells. HCT116 cells were grown in 10 cm dishes for 24 h to 30-50 % confluency and then treated with Lipofectamine RNAiMAX transfection reagent (10 µl) in the presence of 800 pmol non-targeting (NT) siRNA or TRIM26 siRNA for 72 h. Cells were also grown in 10 cm dishes for 24 h to ~90 % confluency and then treated with Lipofectamine 2000 transfection reagent (10 µl) in the presence of a mammalian expression plasmid for Flag-tagged NTH1 (500 ng) for 24 h. Whole cell extracts were prepared and analysed by 10 % SDS-PAGE and immunoblotting with antibodies raised against NTH1 (**A** and **B**; ab70726 and sc-367354, respectively) or (**C**) Flag-tag. Unfortunately the NTH1 antibodies (ab70726) generate a non-specific protein band that migrates in the same position as that of exogenously expressed Flag-tagged NTH1. However whilst the NTH1 antibodies (sc-367354) are of very poor quality, a band of increasing intensity at the expected molecular weight (highlighted in red) is observed in cells overexpressing NTH1. The level of NTH1 protein overexpression does appear to be similar to that of the endogenous protein.
